# Determination of maize hardness by biospeckle and fuzzy granularity

**DOI:** 10.1002/fsn3.130

**Published:** 2014-06-02

**Authors:** Christian Weber, Ana L Dai Pra, Lucía I Passoni, Héctor J Rabal, Marcelo Trivi, Guillermo J Poggio Aguerre

**Affiliations:** 1 Facultad de Ciencias Agrarias y Forestales, Universidad Nacional de La PlataCC31 Correo Argentino, La Plata, 1900, Argentina; 2 Comisión de Investigaciones Científicas de la Provincia de Buenos Aires (CIC-PBA), Centro de Investigaciones Ópticas (CONICET La Plata-CIC)P.O. Box 3, Gonnet, La Plata, 1897, Argentina; 3 Grupo de Inteligencia Artificial aplicada a Ingeniería, Departamento Matemática, Facultad de Ingeniería, Universidad Nacional de Mar del PlataJuan B. Justo 4302, Mar del Plata, 7600, Argentina; 4 Laboratorio de Bioingeniería, Departamento Electrónica, Facultad de Ingeniería, Universidad Nacional de Mar del PlataJuan B. Justo 4302, Mar del Plata, 7600, Argentina; 5 Centro de Investigaciones Ópticas (CONICET La Plata-CIC) and UID Optimo, Departamento de Ciencias Básicas, Facultad de Ingeniería, Universidad Nacional de la PlataP.O. Box 3, Gonnet, La Plata, 1897, Argentina

**Keywords:** Biospeckle, dry milling, laser, maize hardness

## Abstract

In recent years there has been renewed interest in the development of novel grain classification methods that could complement traditional empirical tests. A speckle pattern occurs when a laser beam illuminates an optically rough surface that flickers when the object is active and is called biospeckle. In this work, we use laser biospeckle to classify maize (*Zea mays* L.) kernel hardness. A series of grains of three types of maize were cut and illuminated by a laser. A series of images were then registered, stored, and processed. These were compared with results obtained by floating test. The laser speckle technique was effective in discriminating the grains based on the presence of floury or vitreous endosperm and could be considered a feasible alternative to traditional floating methods. The results indicate that this methodology can distinguish floury and vitreous grains. Moreover, the assay showed higher discrimination capability than traditional tests. It could be potentially useful for maize classification and to increase the efficiency of processing dry milling corn.

## Introduction

The worldwide use of maize under a variety of climatic, edaphic as well as anthropologic situations permits an extraordinary variability in the harvested product.

Currently it is one of the most important cereals cultivated on a global scale. The principal producers are the United States, China, Brazil, México, India, and Argentina (USDA 2013).

Of the total production, about 63% is destined as a feed ingredient for animal rations, while 26% is used for human consumption and the remaining 11% for industry (FAO 2003).

Maize grains are used in about a thousand industrial applications: starch, flour, meal, gluten, oil, paper, adhesives, alcoholic beverages, cosmetics, etc. (Watson 1983). For each use, its quality is associated both with its physical constituents that determine texture and hardness and with its chemical components that define the nutritional value and technologic properties (Gaytán Matrínez et al. 2006). A maize kernel is constituted by four principal parts, where the endosperm represents 80–85%, the embryo 10–12%, pericarp 5–6%, and aleurone 2–3%. Carbohydrates are the principal components of maize kernels, mainly starch (75%), proteins (9%), and lipids (3.5%). Some small quantities of fibers, sugars, minerals, and vitamins are also present (Berger and Singh 2010).

The chemical composition of the endosperm determines different grain shapes and its physical characteristics define commercial types. The most typical commercial types of maize are *hard* or *flint* and *dent*. Nevertheless, some industries typify a third category as *half*-*dent*. *Flint* maize type (*Zea mays* var. *indurata*) present grains with a predominantly vitreous (Wu 1992) nature. They have a low component of starch and high protein content that gives a corneal appearance to the endosperm. The grain edges are shaped as round crowns and present good behavior for harvesting, transfer, and storage. For analytic purposes, those that do not present indentation are considered *flint*; in a longitudinal cut their endosperm macroscopically shows a central floury region surrounded by a corneous one (Brown et al. 1985). They must have a maximum value of 25% in the floating test and a minimum hectoliter weight of 76 kg/HL to fulfill the standard.

On the other hand, maize is classified as dent type (*Zea mays* var. *indentata*) (SAGPyA 2008) when it has a starchy nature and presents pronounced indentation in the crown. In this type, the endosperm is characterized by a high proportion of starch and low protein content. Due to this fact, in its postphysiologic maturity phase as it loses humidity, an indentation appears in the crown giving it a tooth-like appearance (Brown et al. 1985).

Kernel hardness is an intrinsic property of the maize, with a commercial value that is expressed in its resistance to mechanical action and is related to the presence of corneal endosperm, thus presenting higher density. This characteristic is very important in dry-milled processing, where a range of flours and grits are produced. A variety of indirect measurements are highly correlated with hardness have been found (Pratt et al. 1995; Blandino et al. 2010). For this reason, at present there is no standard measure for determining the grain type and quantifying its parts that permits efficient classification of the industrial material. This implies possible losses in industrial procedures due to low yield of the raw material.

The floating test in a sodium nitrate (NaNO_3_) solution is a current analytical procedure used to determine maize grain types in Argentina (Robutti et al. 2000). It is based on the principle that hard grains (flint) are denser and float to a lesser degree than those with lower density (floury or dent). This method is used to compare densities in different maize batches. This methodology is closely related to others such as the grain test weight that is used in the United States (Pratt et al. 1995; Blandino et al. 2010). The floating grains quantification does not permit a determination of the proportions of floury and vitreous endosperm in the grains of a certain sample.

**Figure 1:**
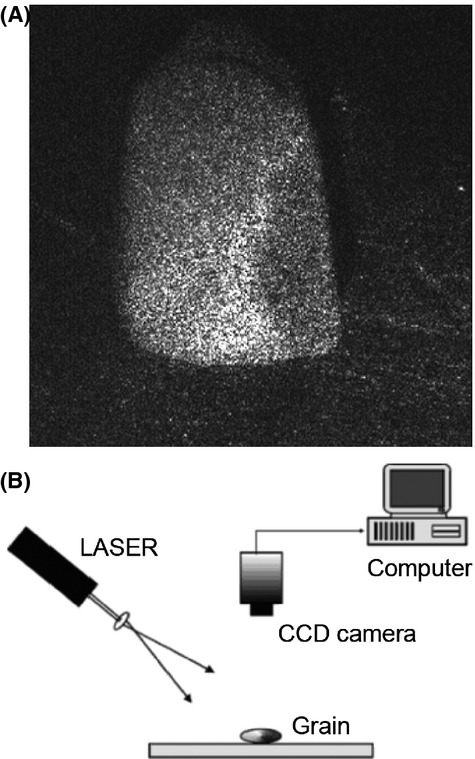
(A) Speckle on a corn seed. (B) Experimental setup used to record and register dynamic speckle images of maize grain.

In this work, we explore the possibility of establishing these proportions using a laser-based optical method named speckle. A speckle pattern (Fig. 1A) is the characteristic light distribution produced when a laser beam illuminates an optically rough surface (Dainty 1975). It consists of a random set of bright and dark points due to optical interference between wavelets originating at different points on the surface and traveling different optical paths to the detector.

In cases of nonstatic surfaces, the speckle patterns change along with time. Typically this phenomenon is present when a laser light illuminates biological tissues and is named “biospeckle” (Braga et al. 2003; Pomarico et al. 2004; Rabal and Braga 2008). Due to the different porosities present and consequently variations in the evaporation capacity of the water contained in the wet samples, the speckle pattern changes over time (Zimnyakov et al. 2001, 2009).

For example, the floury and vitreous regions of a kernel are expected to evaporate water at different rates, so different speckle activities in different regions of a grain are therefore expected according to its local porosity.

In this work, we evaluated speckle activity (biospeckle) measurement to estimate the proportions of vitreous and floury fractions in a set of maize grains in order to identify three commercial types (dent, half-dent, and flint). The results are then contrasted against the floating test.

## Materials and Methods

Maize kernels were provided by the la *Asociación de Cooperativas Argentinas* (ACA) in three sample sets identified by their genetic origin as follows:

1. *Flint* (ACA 929)
2. *Dent* (ACA 467)
3. *Half-dent* (ACA 926)

### Floating test

The tests were performed in the Laboratory of Cerealiculture of the Agronomy and Forestry Faculty at La Plata National University (UNLP).

To determine the type of maize by means of the floating test, the Protocols in Standard 29 of the Argentinean Ministry of Agriculture (MinAgri, ex SAGPyA) were followed.

A 170-mL aqueous solution of NaNO_3_ was prepared, obtaining a density of 1.25 relative to water. It was preserved at 35°C. The next day, when the solution was stabilized, subsamples were measured. This process was performed to control the density and temperature of the solution between measurements.

In all the subsamples (100 grains) the percent humidity was stabilized at 14.5%. They were placed in the solution and stirred with a glass stirrer for 5 min, every 30 sec, to eliminate air bubbles; floating grains were then separated and counted.

The flotation index (*FI*_s_) per sample was then calculated as:


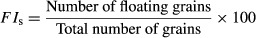
(1)

where *FI*_s_ is the percentage of floating grains with respect to the total number of grains in the sample. The test was repeated five times with three subsamples of each different type of corn and the mean value of the *FI*_s_ excluding the two extreme values (standard 29 SAGPyA) was computed.

### Laser biospeckle

The optical tests were performed in the Laboratory of the Centro de Investigaciones Ópticas (CIOp CONICET La Plata-CIC).

To perform this method, subsamples of maize grains (10 of each type) were wetted during 12 h. They were then cut longitudinally and illuminated with an expanded attenuated He Ne laser beam (10 mW and 632 nm wavelength). A CCD camera (Pulnix TM—6CN CCIR; PULNiX America, Inc., Sunnyvale, CA, 8.6 × 8.3 *μ*m pixel size) was used to register the images. They were digitized by a frame grabber and stored and processed by a personal computer.

A series of 400 images of each subsample were grabbed and stored with 8 bit intensity resolution and 512 × 512 pixels. The sample frequency was about 1 Hz. Figure 1B shows a sketch of the experimental setup.

A series of different corn types classified as *flint*, *dent*, and *half-dent* were analyzed. Ten grains from each subsample (*flint*, *dent*, and *half-dent*) were screened. How each pixel evolved in the image sequence was observed. Each evolution was considered a time series and digitally processed. In this way we distinguished regions with different activity levels.

The time analysis of a dynamic speckle signal provides information about the phenomenon that produces the microscopic movements in each grain. The descriptors used to analyze signals can also be applied to image sequences.

In this way, we obtain a descriptor value for each pixel and its variations in space that can be used to screen spacial regions with different activities in the same speckle pattern.

### Fuzzy granular descriptor

This descriptor is an abstraction of the granular appearance of the speckle phenomenon; it is intended to quantify the number of times that a pixel changes intensity in a fixed time period (Pedrycz and Gacek 2002; Dai Pra et al. 2009).

In granular computing, the information is grouped in entities that fulfill conditions of similarity. One of its purposes is the need to process information in a human-centric modality, in this case being the granules, conceptual entities that emerge as a direct consequence of the quest to identify abstract objects and their processing (Zadeh 1970; Pedrycz 2001).

The fuzzy sets facilitate the interpretation of concepts with indefinite limits. They are characterized by a membership function that assigns to every element of the set a membership degree in the [0,1] interval (Zadeh 1965).

Given a universal set *U* of elements *x*_*i*_, a fuzzy set *A∈U* is defined by pairs of elements (*x*_*i*_*, μ*_*A*_(*x*_*i*_)), where *μ*_*A*_(*x*_*i*_) is a real value in [0,1] that represents the membership degree of *x*_*i*_ to *A*.

In intensity pixel sets, *U* is represented by the intensity values 0–255 and the fuzzy sets are represented by concepts such as *dark*, *medium*, and *light*, which are intervals of intensity values. They are subjective, overlapping, and with undefined limits (Dai Pra et al. 2009). The granularity methodology tries to quantify the times that a pixel changes intensity over a fixed time period by considering the fuzzy concepts *dark*, *medium*, and *light* instead of the numerical intensity values.

### Determination of the vitreous and floury fractions in the processed images

**Figure 2:**
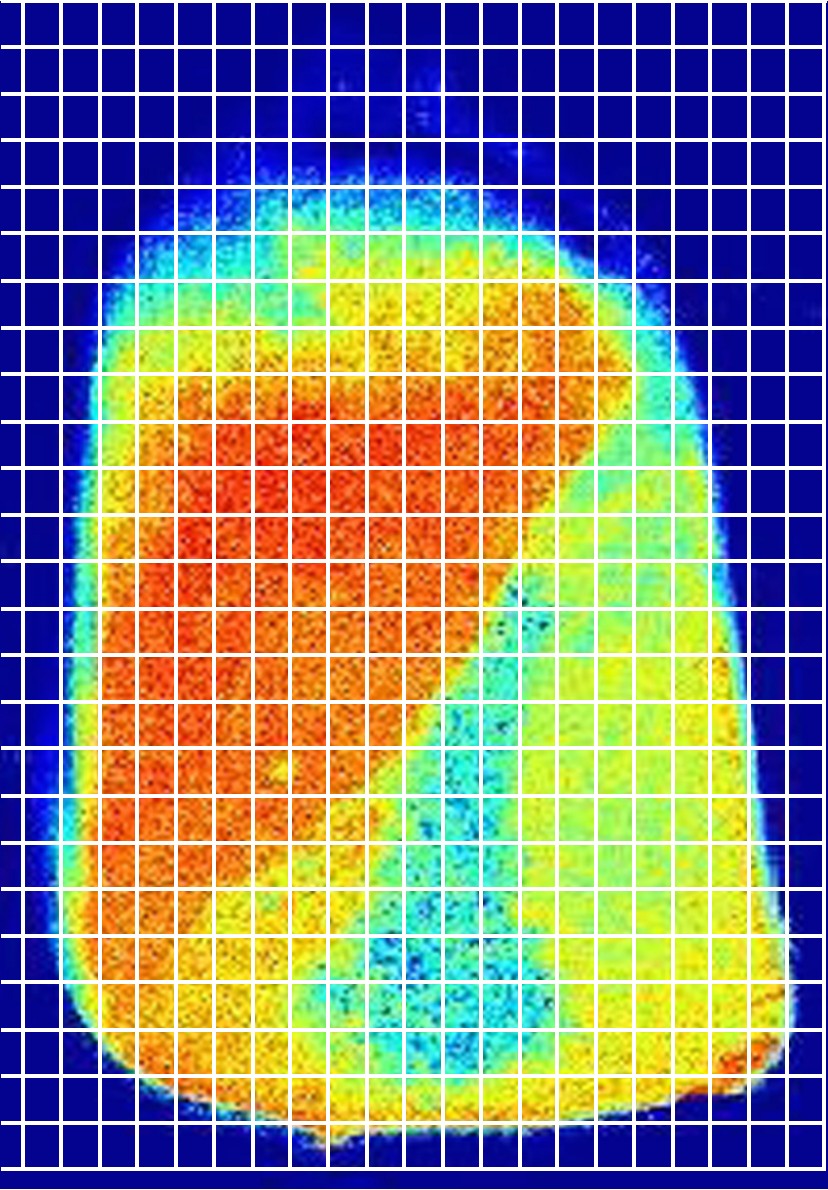
Processed image divided into grid cells for counting the fraction of each grain component: orange for the embryo, light blue for floury endosperm, and yellow for vitreous endosperm.

The method used in this work to classify the different components is not still fully automated. In the images processed as described before, each component was identified by its color and a fraction (vitreous or floury) was assigned based on knowledge of grain anatomy. This procedure was followed as an adaptation of other visual methods that have been cited as effective in this identification, such as the hard–soft endosperm ratio (H/S) (Blandino et al. 2010). To that end, a grid was superposed on each image as shown in Figure 2 (Lutman 1992; McCollum 2005). In our case, each image was divided into 25 × 22 cells, in which the presence of all the components of the grain (including the embryo) was determined.

To that end, each cell in the grid was subjectively classified depending on the color of the processed image. Based on knowledge of the grain morphology, it can be seen that the majority of grain components show different colors. The embryo stands out in red, the vitreous endosperm in the external region appears yellow-orange, and the central part of the floury endosperm appears as pale blue-green. These differences in speckle activity are mostly due to divergent capacities to lose water in regions with dissimilar porosity. The number of classified cells on each grid was stored in counters (one counter for the embryo, one for the vitreous fraction, and one for the floury one).

Then, the results stored in the counters were added and the proportion for each image was calculated. This procedure was double blind and repeated three times, without knowing the results of the previous observations.

## Results and Discussion

### Floating test

**Figure 3:**
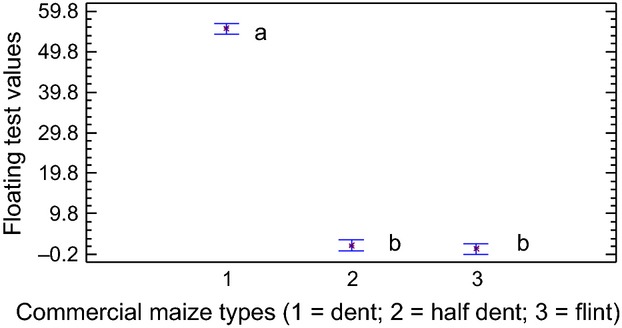
Graphic of means showing the flotation test results based on maize type. Different letters indicate significant differences according to low statistical difference (LSD) test (*P* = 0.05).

Initially, data from the floating test were collected and are shown in Figure 3. It can be seen that the measurements obtained for each grain type showed a wide difference between samples classified as dent (56%) with respect to half-dent (2%) and flint (1%) samples.

By means of this test, it was observed that more than 50% of the grains corresponding to the dent type floated in the solution. This is due to the fact that dent grains have a higher percentage of floury endosperm in their composition, which results in a lower density (Mestres et al. 1991).

The other two types (half-dent and flint) showed relatively few floating grains in the solution, no more than 3%. This indicates an endosperm conformation that is substantially different from the dent type, as shown by the higher density of these types. This may be the reason why the floating test cannot correctly classify the three types. As is obvious, the floating test includes all grain components, such as the embryo, that contribute to the final grain density and consequently its ability to float or not.

Nevertheless, no differences could be found between the densities of these latter groups that would permit estimation of contrasts in the quantitative composition of their endosperms. This was confirmed by the results of analysis of variance (ANOVA) tests between the different types of commercial corn (as factors) and the flotation index (as a variable) (Fig. 3).

We verified the degree of significance by means of the low statistical difference (LSD) test and it showed two clearly distinguished groups (*P* < 0.05). One of them comprised the dent type and the other by the two remaining types. These latter types could not be resolved (Fig. 3).

### Biospeckle test

**Figure 4:**
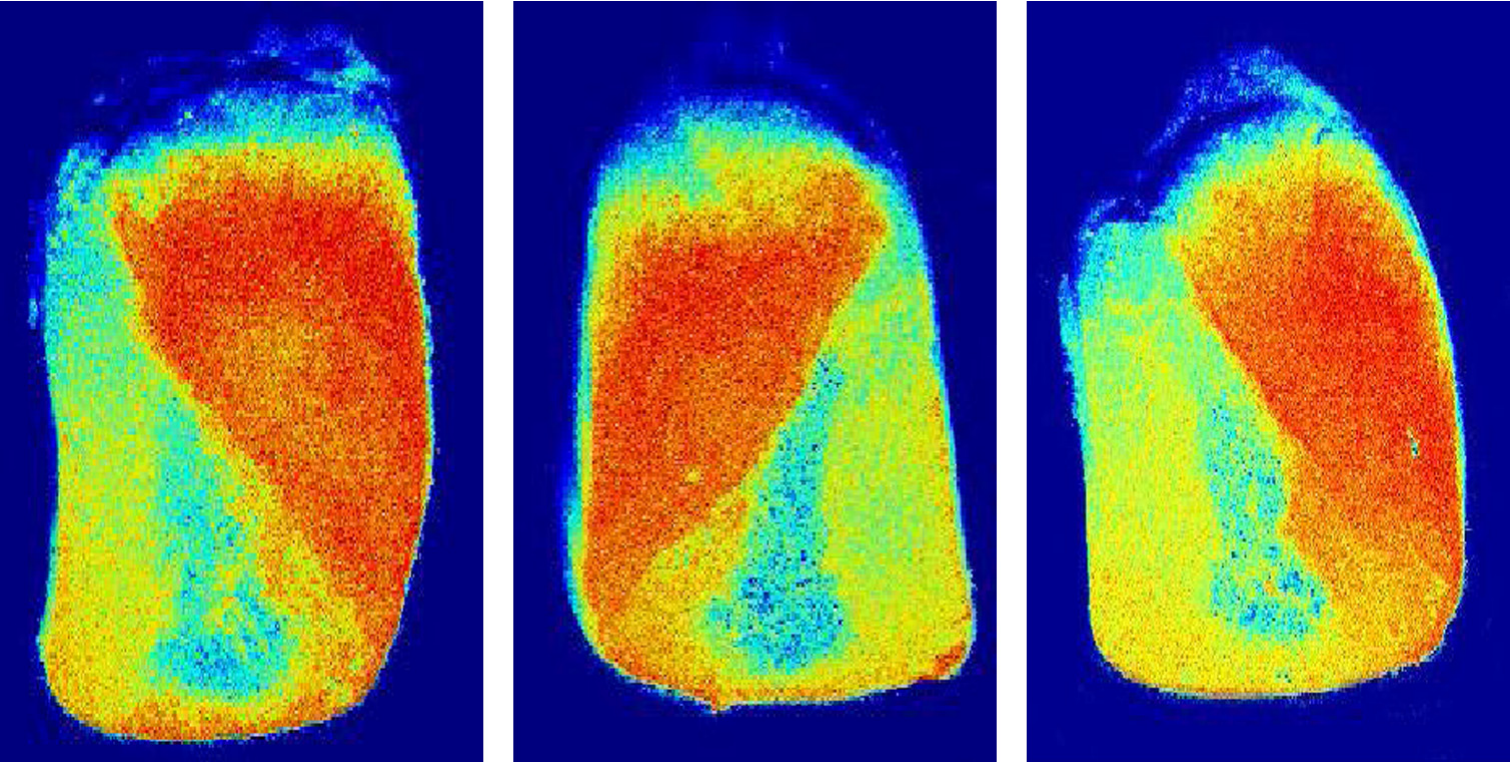
Example of three transversely cut maize grains. Pseudocolored images were obtained from a series of speckle images after processing by the Fuzzy method.

Then we proceeded to compare the respective biospeckle images of the different samples processed by means of a MATLAB® (The MathWorks, Inc., Natick, MA) program using the Fuzzy method, as shown in Figure 4. In this figure, three sample pseudocolored images obtained from a series of speckle images after processing by the Fuzzy method are shown. This method was selected based on the improved contrast and definition observed when compared with other descriptors.

**Figure 5:**
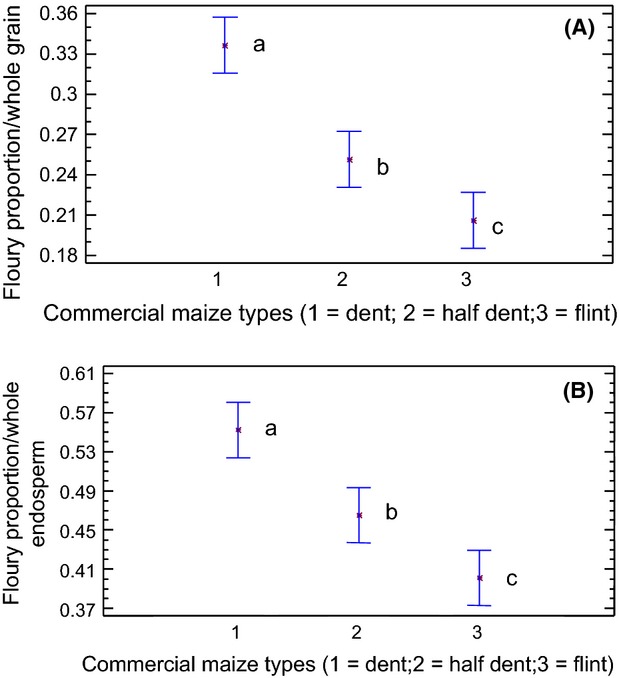
(A) Graphic of means showing the floury/whole grain ratio of different maize types as measured by the biospeckle method. Different letters indicate statistical differences as obtained with the low statistical difference (LSD) test (*P* = 0.05). Bars indicate maximum departures from the mean. (B) Graphic of means showing the relationship between different maize types and means of the floury/whole endosperm ratio as measured by biospeckling. Different letters indicate significant differences as given by the LSD test (*P* = 0.05).

The results for the floury endosperm/whole grain ratio and the floury endosperm/whole endosperm ratio obtained with the grid method applied to the images processed with fuzzy granularity are shown in Figure 5A and B. Notice that, by applying the speckle method, the higher proportion of floury endosperm in dent-type corn compared with the half-dent and flint groups is apparent. These results were not apparent using the flotation method.

The mean floury endosperm/whole endosperm proportion for dent-type maize was 0.55. This result is in accordance with the flotation test results. Nevertheless, the values obtained for the other two groups: 0.43 and 0.41 (half-dent and flint, respectively) were not resolved by the flotation test classification.

The obtained results agree with the higher proportion of floury endosperm present in the dent grains, which reduces its density. The protein matrix and starch grains in this fraction determine a higher proportion of porous regions (Robutti et al. 1997). In half-dent and flint types, significant differences were found in the proportion of floury endosperm (*P* < 0.05), unlike what was found in the flotation test. As shown in Figure 5A, the half-dent type had a higher floury endosperm/whole grain proportion than the flint type. This difference was not significant with the flotation method.

The same behavior was observed when the floury/whole endosperm proportion was considered (Fig. 5B).

In the same way we obtained the proportion of vitreous endosperm with respect to the whole grain and the proportion of vitreous endosperm in the whole endosperm.

**Figure 6:**
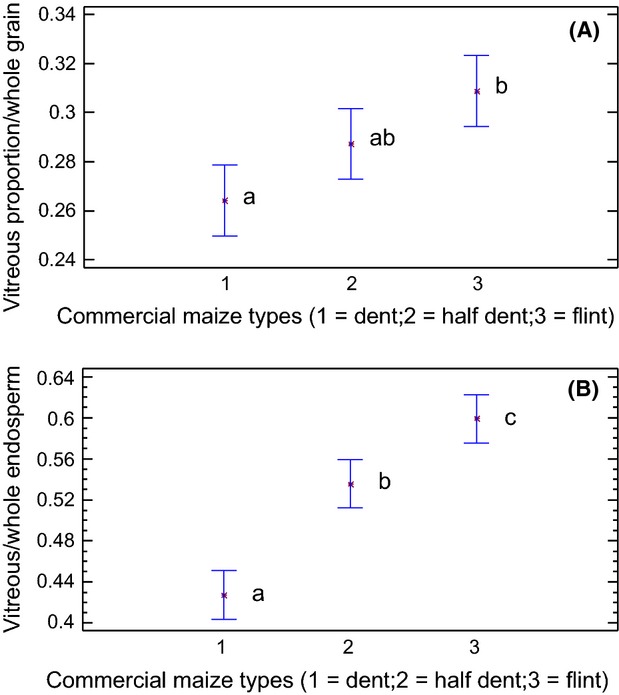
(A) Graphic of means showing the relationship between different commercial maize types and the vitreous/whole grain ratio with the biospeckle method. Different letters indicate statistically significant differences as per the low statistical difference (LSD) test (*P* = 0.05). (B) Graphic of means showing the relationship between different maize types and the vitreous/whole endosperm ratio. Different letters indicate statistically significant differences as per the LSD test (*P* = 0.05).

No perceivable differences in the means of the percentage of vitreous in the whole grain were found (0.273, 0.287, and 0.309 for dent, half-dent, and flint, respectively) (Fig. 6A). Nevertheless, when the vitreous/whole endosperm proportion was analyzed, significant differences were obtained (0.452, 0.544, and 0.596) (Fig. 6B).

The results concerned with the proportion of vitreous to whole endosperm did show significant differences in the three groups, as shown in Figure 6B.

The results obtained with the floating test using dent (0.55) and flint (0.01) maize types are within the typical values established by current Argentine standards (as in Argentine Agriculture Secretary Doc. No. 1075/94). The half-dent type is not considered by this Secretary.

As for the results obtained by the speckle measurements, the proportions of floury and vitreous in the whole grain and the whole endosperm were within the theoretical percentage limits corresponding to the analyzed composition of each grain. The half-dent type could be determined because it presented intermediate values between the other two types.

The evaluated method could permit a more detailed classification of grain types. This may be required when the maize is intended for different industrial processes and could increase its added value.

The supposition initially evaluated in this work has been supported by experiment using an optical method on the analyzed images, where values corresponding to the proportions of floury and vitreous endosperm in all the analyzed grains could be determined. They were positively confirmed by information provided by the ACA with respect to the genetic precedence of the material provided for the performed study.

### Automation prospects

Although the speckle results still need more detailed research and instrumentation in order to obtain full automation, we made a preliminary test with the flint type.

**Figure 7:**
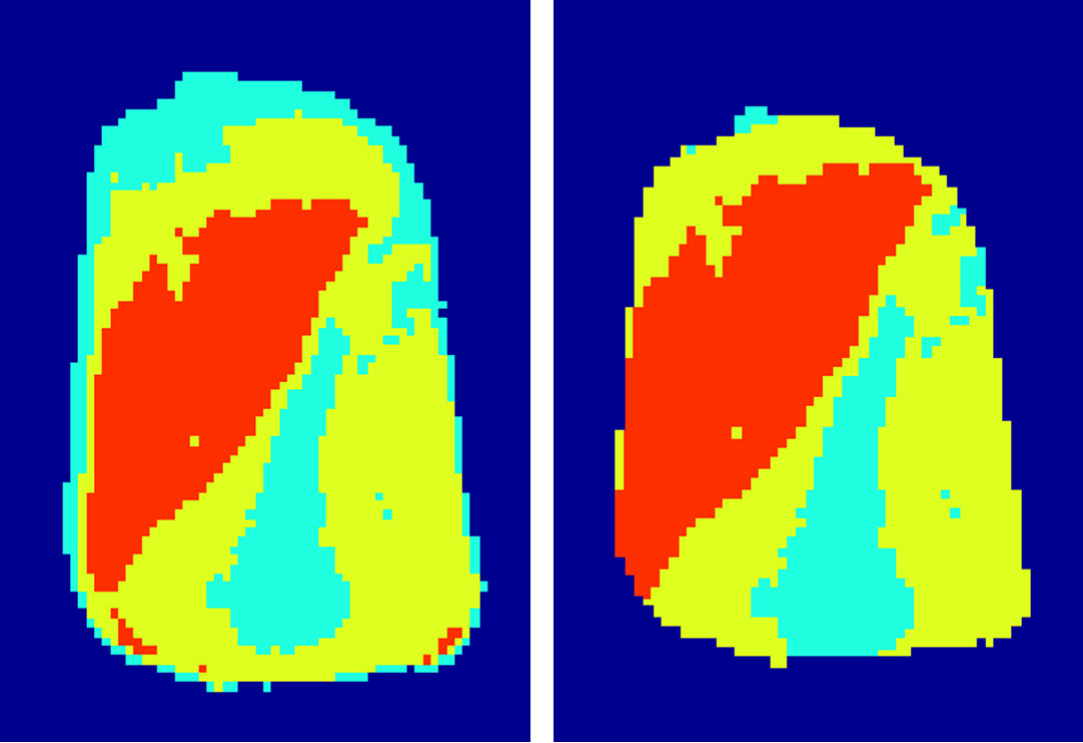
Images resulting from the automated method. Orange, embryo; light blue and yellow, floury and vitreous endosperm, respectively.

The methodology consists of applying mean values to slide windows of the processed image and then painting the corresponding pixels according to defined thresholds. When the threshold values are selected as a function of the predominant colors in the processed image (orange, yellow, and light blue), an image as in Figure 7A is obtained.

As the processed image has soft edges due to the grain shape, a process of cutting around the active zones is then applied. It is made by expanding the inert zone (blue region) as shown in Figure 7B. In the last image the proportion of each area is evaluated.

This methodology shows promising results compared with results obtained visually using the grid method. Small differences between the two results are shown in Table 1.

**Table 1:** Mean values of vitreous endosperm and floury/whole grain proportions, and vitreous endosperm and floury/whole endosperm proportions obtained using laser biospeckling on dent-type maize by grids and by an automated method.

Determination mode	Vitreous endosperm/whole grain	Vitreous endosperm/whole endosperm	Floury endosperm/whole grain	Floury endosperm/whole endosperm
Visually by grids	0.309	0.599	0.224	0.406
Automatically by processing	0.379	0.638	0.217	0.362

We are currently testing how to improve the performance of the automated method by the simultaneous use of different descriptors appearing in the literature.

The comparative optical results with the floating test are very interesting because the latter test has been reported to correlate highly with at least other six maize hardness determination tests. In addition, this new method would permit the classification of grains with respect to their endosperm composition and proportions of different constituents, with higher sensitivity compared with traditional methods. Specifically, with the H/S method the speckle is more accurate, given that the activity of each component is specific to each one.

## Conclusions

The results obtained in this work confirm that it is possible to use laser biospeckling to reliably determine the proportions of vitreous and floury in maize grains.

Comparative advantages of the optical method versus the floating test are that the former permits differentiation of the three major tested commercial types (dent, half-dent, and flint) while the floating test did not permit that differentiation.

The determinations obtained with the optical method could be extended to other commercial corn types as well as to other cereals.

On the other hand, the tested method permits to determine the proportion of embryo (not provided by the flotation method). This proportion then can be discounted to find the actual endosperm fractions.

It also provides a result expressed as a number (quantitative results). It could be better than the qualitative classification given by floating test.
